# Spectroscopy-Based Cell Culture Predictive Monitoring

**DOI:** 10.3390/biotech15020035

**Published:** 2026-05-20

**Authors:** Ahmed Kanfoud, Pascal Gerkens, Marie Bastin, Laurent Rondia, Florian Ceulemans, Karim Donnay, Bertrand Debuisseret, Thomas Cornet, Gael de Lannoy, Thibault Helleputte

**Affiliations:** 1 DNAlytics, 1 Rue Jean Monnet, B-1348 Ottignies-Louvain-la-Neuve, Belgium; 2 GlaxoSmithKline Biologicals SA, Rue de l’Institut 89, B-1330 Rixensart, Belgium

**Keywords:** cell culture, predictive monitoring, process analytical technology

## Abstract

Spectral monitoring combined with chemometrics models resulting from machine learning approaches allows cell culture to be monitored almost in real time. This process analytical technology offers to drastically reduce the amount of hands-on time and laboratory testing needed to monitor this crucial biomanufacturing step. In this article, we propose a method to anticipate future spectra. The method is based on extrapolation of the spectra in a reduced-dimensionality space, followed by retroprojection in the original space. Passed to regular chemometrics models already fitted, these anticipated spectra enable predictive cell culture monitoring up to several dozen hours with satisfactory quality. This anticipation paves the way for course-correction and enhanced operations such as reduced need for night shifts.

## 1. Introduction

Spectroscopic techniques are widely recognized as cornerstones of Process Analytical Technology (PAT), a framework established for the real-time monitoring and control of industrial processes [1]. Raman spectroscopy knows a growing adoption in the healthcare industry, associated sensors becoming more and more fit for practical use [2,3]. In the context of biomanufacturing, these techniques are pivotal for gaining a deeper understanding of production dynamics and are fundamental to the development of new products [4,5,6,7]. The goal of PAT is to build quality into products by design, moving away from reliance on end-product testing towards a more proactive, science-based approach to process control [8,9]. However, new product development remains a notoriously lengthy and resource-intensive endeavor. The adoption of PAT-based tools, such as in-line spectroscopic probes, provides a powerful approach that can better manage these efforts [4]. A key advantage lies in moving beyond simple monitoring towards advanced strategies like Model Predictive Control (MPC), which uses process models to anticipate future outcomes [10,11,12,13]. Recent work on data-driven predictive modeling for commercial cell culture processes further demonstrates how at-line and on-line measurements can be used to predict critical performance attributes several days in advance, supporting proactive decision-making in biotherapeutic manufacturing [14]. For instance, predicting the final state of fermentation allows for a wider range of conditions to be tested more efficiently. This enables the early termination of unpromising culture batches, freeing up valuable equipment faster and significantly accelerating project timelines. This predictive capability is particularly crucial in the early stages of product development, where historical data is limited and only a few batches have been completed. In such scenarios, building robust, traditional chemometric models is often not feasible due to the scarcity of comprehensive datasets [15] and the cost of generating large calibration sets [1,16,17]. Therefore, a significant advantage would come from a predictive method that is quick to implement and does not require specialized expertise in machine learning or an extensive historical database. Such an approach would unlock early process insights and accelerate optimization efforts at the most critical phase of development. This paper introduces a method that directly addresses this challenge, enabling early and reliable predictions in cell culture monitoring without the need for a pre-existing model. The proposed method does not rely on the very long and complex establishment of a mechanistic model, nor does it rely on machine learning-based techniques generally requiring a number of pre-existing and annotated batches that is often larger than the context of process development allows. This method allows us to rely solely on the spectra gathered from the early hours of the culture and extrapolate future spectra within a projected, simplified mathematical space before backprojection in the original space of the spectra. This is explained in Section 2. This section also introduces evaluation metrics and actual data used to illustrate and validate the method. Section 3 puts the method in practice on actual vaccine manufacturing data. Section 4 discusses the soundness, the usefulness and perspectives of extensions of the method.

## 2. Materials and Methods

### 2.1. Data

#### 2.1.1. Strain and Culture Conditions

A proprietary recombinant yeast strain of Saccharomyces cerevisiae was used in this work. The fermentations were conducted at GSK (Rixensart, Belgium). Each culture was initiated in a bioreactor with a starting volume of 5.5 L. The regular process temperature setpoint is a constant 30 °C, and the regular pH setpoint is at 5.0 through. This level can be maintained via the controlled addition of a base solution. Dissolved oxygen ($pO_{2}$) is monitored using an in-line sensor, and the stirrer speed, initially set at 260 rpm, can be automatically adjusted to maintain the $pO_{2}$ level above 60% of air saturation. The first 20 h are achieved in batch mode, followed by a fed-batch culture.

To induce sufficient variability within the dataset of this study, we modulated key process parameters that are known to influence cell metabolism and growth. Specifically, this included aeration conditions (different dissolved oxygen setpoints were tested across the runs) and nutrient feeding profiles (different glucose feeding strategies have been applied) to induce a range of metabolic states throughout the fermentations. The level of variation is sufficient to reflect what is traditionally done in a process development setting, i.e., it is much more significant than the amount of variation that would be witnessed in a routine manufacturing setting.

The cultures were conducted at GSK, Rixensart, Belgium.

#### 2.1.2. Experimental Design

A total of twenty-three fermentation lots were performed for this study, each running for approximately 90 h. To build a robust and varied dataset, these fermentations were conducted using different process parameters and included targeted spiking of key metabolites.

#### 2.1.3. In-Line Raman Spectroscopy

In-line process monitoring was carried out using a Kaiser Raman RXN2 system equipped with a probe immersed in the bioreactor, similar to previously reported applications of Raman PAT in fermentation processes [1]. The system was configured to collect spectra across a range of $100\;{\mathrm{cm}}^{-1}$ to $3425\;{\mathrm{cm}}^{-1}$. For the duration of each fermentation, a Raman spectrum was acquired every hour. The acquisition time for a single spectrum was 5 min.

#### 2.1.4. Offline Sampling and Reference Analysis

Synchronized with each hourly Raman measurement, an offline sample was manually collected from the bioreactor. These samples were immediately analyzed to determine the biomass concentration via optical density measurement at 650 nm (${\mathrm{OD}}_{650}$).

${\mathrm{OD}}_{650}$ was measured on a spectrophotometer using diluted samples to ensure that the readings remained within the linear range of the instrument. Because dense yeast suspensions rapidly exceed the linear response range of a standard 1 cm pathlength measurement, samples were diluted in PBS prior to analysis. The reported ${\mathrm{OD}}_{650}$ values correspond to the undiluted culture, and were obtained by multiplying the measured value by the applied dilution factor. As a result, the relatively large OD values reported in this study reflect high biomass concentrations after back-calculation, rather than a direct single-pass absorbance measurement in a 1 cm cuvette.

Across all twenty-three fermentations, a total of 1580 paired in-line spectra and offline reference samples were collected.

### 2.2. Spectra Anticipation Method

**Figure 1:**
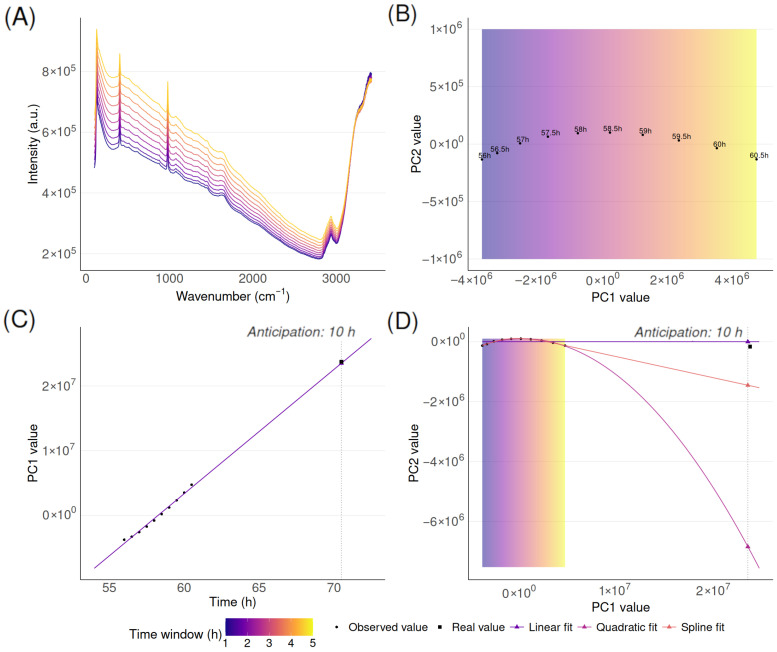
Accumulated spectra and PCA representation for a 10 h anticipation horizon. (**A**) Accumulated spectra in original space. (**B**) Accumulated spectra in PCA space. (**C**) PC1 against time. (**D**) PC2 as a function of PC1 with fitted models. The color gradient represents the time window used for fitting and is shown to emphasize that long-term anticipations are obtained from a limited number of early observations, illustrating the extrapolative nature of the approach.

**Figure 2:**
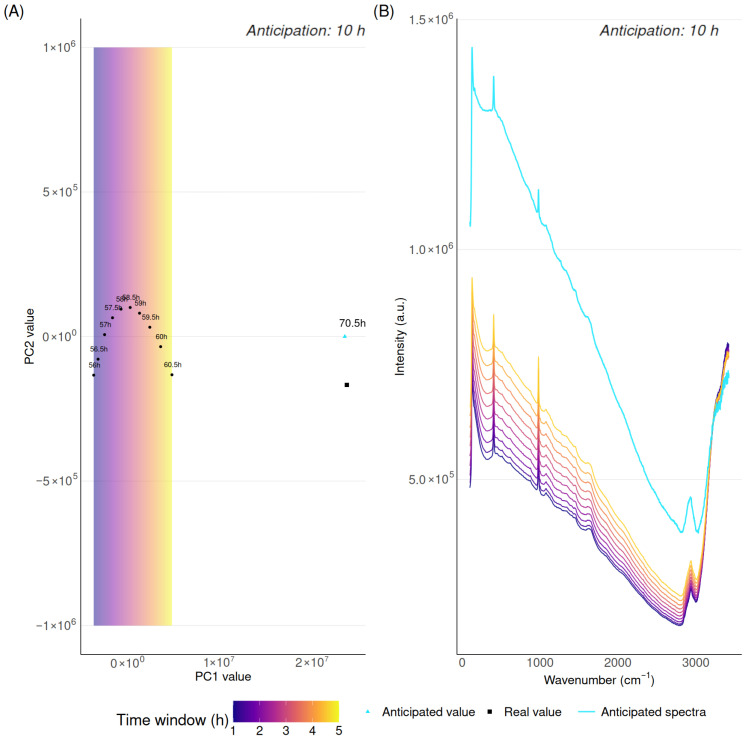
Spectral anticipation based on PCA for a 10 h anticipation horizon. (**A**) Anticipated spectra in PCA space together with accumulated spectra. (**B**) Anticipated spectra together with accumulated spectra back-projected in original space.

The method for spectra anticipation applies independently to each manufacturing batch (i.e., independently for each cell culture run). In summary, it follows an extrapolation strategy, where the spectra generated during the first few hours of the cell culture are used as a basis for anticipating the spectra of the rest of the cell culture. This extrapolation is performed in a lower-dimensional space than the original wavelength space of the signal. Then this lower-dimensionality extrapolation is reprojected back into the original high-dimensional space of the spectra. In more detail, the method consists of four sequential steps that are detailed below. The whole methodology is pictured in Figure 1 and Figure 2.

1. *Accumulation of spectra.* A moving window of 5 h was used to accumulate consecutive spectra from each fermentation batch. This window length was selected as a trade-off, providing a compromise between the number of available spectra and prediction delay. Depending on the acquisition frequency, this window includes 5 to 10 spectra, which was sufficient to ensure stable predictions without requiring long accumulation times.
2. *Projection to lower dimension through Principal Component Analysis.* Principal Component Analysis (PCA) was applied to the spectra accumulated in the considered time window. The first two principal components (PC1 and PC2) were retained. In general, PC1 explained >80% of the variance. The accumulation of just PC1 and PC2 explained 99% of the variance in most cases, which was deemed sufficient.
3. *Extrapolation in projected space.* In the lower-dimensional space (two dimensions) where the early spectra have been projected, we look for future spectra as an extrapolation of the early spectra. We again proceed sequentially: extrapolate along PC1 versus time, then along PC2 versus PC1, taking into account the coordinate along PC1 estimated just before. PC1 values were predicted using linear extrapolation against time. PC2 values were predicted using extrapolation of the PC2 versus PC1 relationship. Linear fitting was selected for both projections.
4. *Inverse transformation to the original data space.* Predicted (extrapolated) coordinates along the two dimensions (PC1 and PC2) were reprojected to the original spectral space to obtain the predicted spectrum. This was done through an inverse application of the PCA projection computed at step 2.

Variants of this method have been studied and compared, playing with starting time, i.e., how soon after cell culture start we can validly extrapolate (0 to 20 h), wavelength range (full, truncated $100\;{\mathrm{cm}}^{-1}$ to $3000\;{\mathrm{cm}}^{-1}$, or selected bands), preprocessing (raw or second-derivative-based detrending), and type of fitting for extrapolation (linear, quadratic, cubic, regularized, …).

This investigation is reported in Appendix A. As a result, the optimal configuration identified is as follows:

- Start time: 20 h from fermentation initiation.
- Wavelength range: full spectrum ($100\;{\mathrm{cm}}^{-1}$ to $3425\;{\mathrm{cm}}^{-1}$).
- Spectrum type: raw (not detrended).
- Spectra accumulation window: 5 h.
- PC1 versus Time: linear fitting.
- PC2 versus PC1: linear fitting.

### 2.3. Baseline Comparator

To provide a reference point for the proposed method, the same evaluation was conducted using a naive baseline model. In this setting, the anticipated spectrum is assumed to be identical to the last observed spectrum available in the observation window, without any predictive component.

### 2.4. Evaluation of the Spectra Anticipation Methodology

The assessment of the proposed method relies on spectra comparison. In particular, we use the cosine similarity $S_{c}\left(A,B\right)$ between a predicted future spectrum (*A*) and an actual, experimental future spectrum (*B*). This cosine similarity, for spectra with dimensionality of *n* wavelengths, is defined as follows:

(1) $$S_{c}\left(A,B\right)=cos\left(\theta \right)=\frac{A\cdot B}{\left||A||\cdot ||B|\right|}=\frac{\sum_{i=1}^{n}A_{i}B_{i}}{\sqrt{\sum_{i=1}^{n}A_{i}^{2}}\cdot \sqrt{\sum_{i=1}^{n}B_{i}^{2}}}$$

Yet, a distance metric alone provides little insight into the usefulness of the method (what is an “acceptable” cosine similarity?). Based on this metric, the evaluation strategy is as follows.

For an anticipated spectrum *j* hours ahead ($Ant_{j}$), all cosine similarities between itself and each of the actually available experimental spectra ($Act_{i}$) within the same cell culture batch are computed, i.e., $S_{c}\left(Ant_{j},Act_{i}\right)\forall i$ but for a single given value of *j*.

Then, the time point $t_{min}\left(j\right)$ for which the cosine similarity is minimal is identified: $t_{min}\left(j\right)=argmin_{i}\left(S_{c}\left(Ant_{j},Act_{i}\right)\right)$. We then compute the difference between this point and *j*, noted $\Delta t_{min}\left(j\right)$ is registered. A method behaving ideally would lead to $\Delta t_{min}\left(j\right)=0$, which can be interpreted as the fact that the most similar actual spectra among all available in the considered cell culture run is the one at the time point for which the spectrum is predicted. Otherwise said, it is desired that $S_{c}\left(Ant_{j},Act_{i}\right)$ is minimal when i=j, and thus it is desired that $\Delta t_{min}\left(j\right)=0$. Otherwise, it is desired that the prediction of the spectrum of 20 h (for example) from now will be closer to the actual spectrum that appeared 20 h later than it is for any other that appeared 5 or 30 h later.

Although more meaningful, the $\Delta t_{min}\left(i\right)$ metric is not common in the literature. The interested reader will be able to find a more traditional evaluation by RMSE in Appendix A.

## 3. Results

### 3.1. Qualitative Assessment

**Figure 3:**
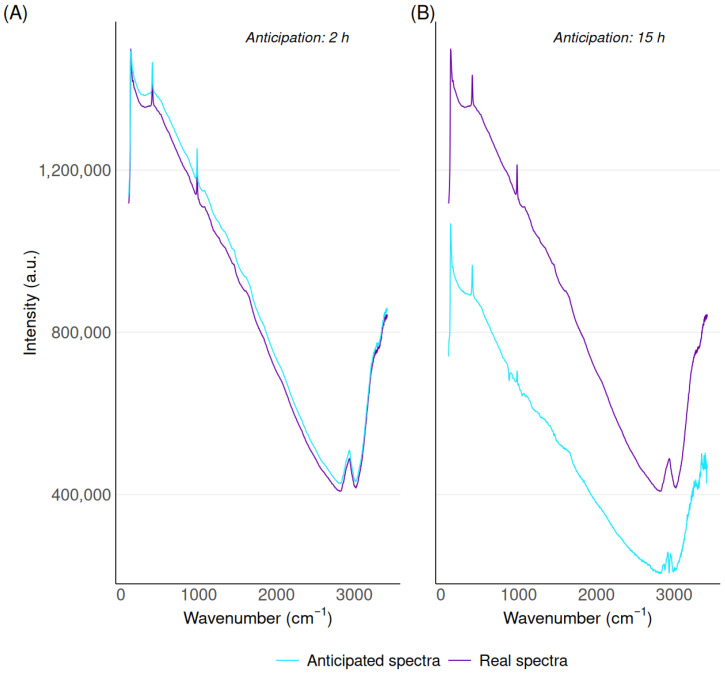
Comparison of anticipated and actual spectra at 2 h (**A**) and 15 h (**B**) ahead.

The evaluation of the method starts with a qualitative assessment. Predictions are generated for periods ranging from 2 to 40 h, and each time compared to the corresponding actual spectrum. Examples with, respectively, 2 h and 15 h of anticipation are depicted in Figure 3. Regardless of the amount of anticipation, specific shapes within spectra are clearly well reproduced (spikes, bell-shaped portions, general trends, …). There is, however, an increase in the discrepancy between anticipated spectra and the corresponding actual spectra as the level of anticipation rises. Although observable, this evolution of anticipation quality with the amount of anticipation does not shed light on the amount of anticipation suitable for practical applications. This will be addressed in upcoming sections as well as in Section 4.

### 3.2. Quantitative Evaluation

**Figure 4:**
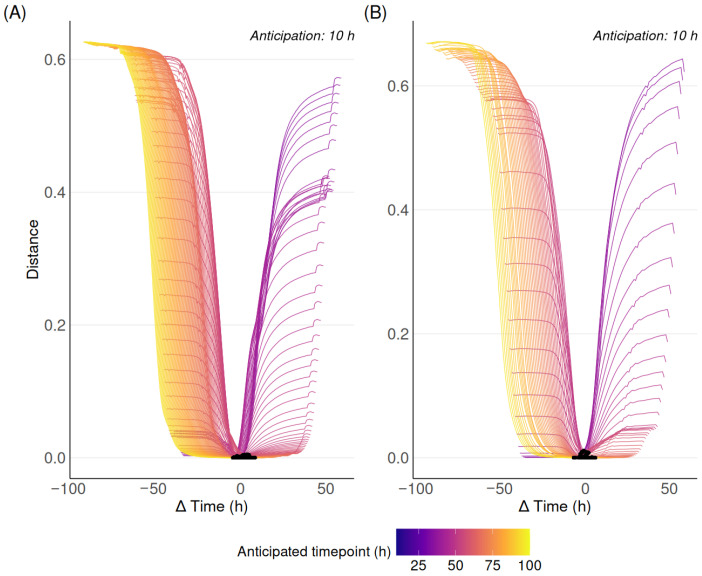
Visualization of the cosine similarity Sc between anticipated spectra (10 h ahead) and all batch spectra. The black dots around 0 at the bottom of the graphs correspond to $t_{min}$. (**A**) Spectra acquired at a 30 min frequency. (**B**) Spectra acquired at a 1 h frequency.

To obtain an objective insight into the method’s performance, we apply the evaluation strategy based on cosine similarity described in Section 2.4. Two examples on two distinct cell cultures are illustrated in Figure 4. The averaged results over all available cell culture runs are reported in Table 1.

**Figure A6:**
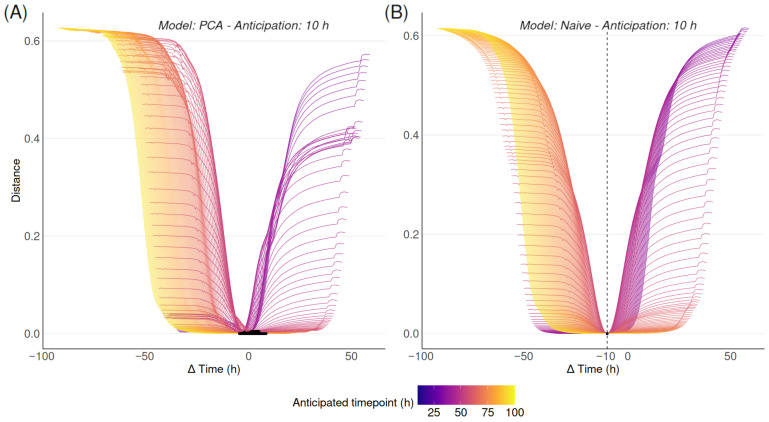
Visualization of the cosine similarity-based distance between anticipated spectra (10 h ahead) and all spectra within the batch for two representative runs. The color scale indicates the anticipated time point. The black markers at the bottom highlight the time of minimum distance ($t_{min}$). (**A**) PCA-based anticipation model. (**B**) Naive baseline model.

The same analysis is also performed with the baseline comparator proposed in Section 2. The results are reported in Table 2 and Figure A6.

The same analysis is also performed after an additional second derivative detrending (d2) is applied prior to the comparison (on both the predicted spectra and actual spectra). The results are reported in Table 3 for the proposed anticipation method, and for the naive baseline in Table A3 of the Appendix A. This additional investigation is motivated by the fact that such detrending is sometimes applied before the application of chemometrics models for metabolite concentration prediction.

Negative $\bar{\Delta t_{min}\left(i\right)}$ values indicate that the closest matching experimental spectrum occurred, on average, before the prediction time point, whereas positive values indicate that it usually occurred after. When considering raw (unpreprocessed) spectra, minimum spectral distances remained close to zero for anticipation horizons up to approximately 10–13 h, beyond which both $\bar{\Delta t_{min}\left(i\right)}$ and its standard deviation increased.

Similar results are obtained when using detrended spectra (d2). No significant difference is observed, although a slight tendency towards lower $\bar{\Delta t_{min}\left(i\right)}$ values over longer anticipation horizons, for both acquisition frequencies. Detrending may thus, on some occasions, improve the temporal consistency between anticipated and actual spectra.

Regarding the baseline comparator, for both preprocessing conditions, $\bar{\Delta t_{min}\left(i\right)}$ exhibits a near-linear decrease as a function of the anticipation horizon *i*, closely approximating −i. This indicates that the closest matching experimental spectrum consistently corresponds to past observations, reflecting the absence of anticipation capability in the baseline approach. In addition, the very low standard deviations observed across all horizons highlight the deterministic nature of this behavior. Again, no noticeable difference is observed between raw and d2-detrended spectra.

An extra comparison is reported in Table A1 and Table A2 of Appendix A, where RMSE and Pearson correlation are reported to compare the naive approach and the proposed spectra anticipation method.

## 4. Discussion

Section 3 measures how anticipated spectra are closer on average to the actual spectra at the considered future time point than they are to actual spectra before and after that future time point. This evaluation rigorously avoids overfitting, as the anticipated spectrum is compared to a spectrum that is not part of the window of spectra used to compute projection, extrapolate in a future time period, and back-project in the original space. The method thus never has access to the actual spectrum it has to anticipate. This evaluation of the method itself, as well as its comparison to a more naive approach, confirm the soundness of the proposed anticipation method. Still, it does not allow to us to draw any conclusions on its usefulness in practice because the distance between spectra is still an abstract notion. To shed additional light on this usefulness, we feed anticipated spectra to a (confidential) ML-based model of biomass for an actual biotech product at GSK [18,19].

**Figure 5:**
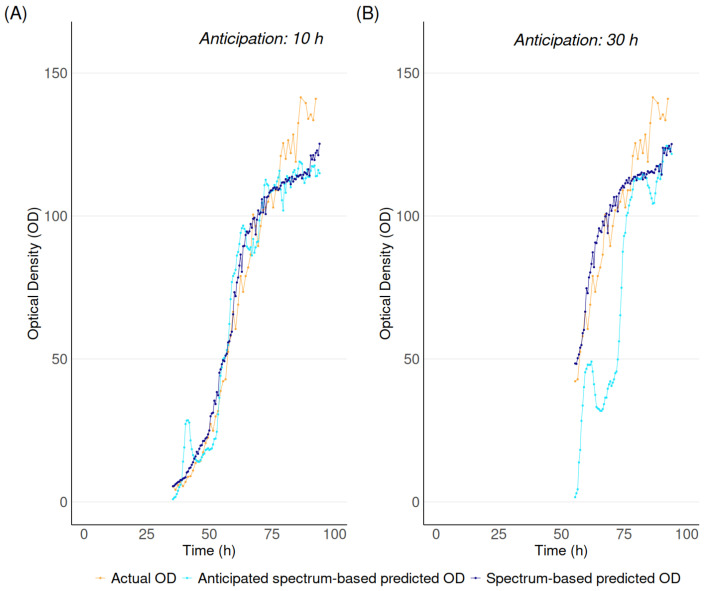
Comparison of actual OD, spectrum-based predicted OD and anticipated spectrum-based predicted OD, 10 h (**A**) and 30 h (**B**) ahead.

Figure 5 graphically compares actual optical density (obtained by wet-lab measurements), spectrum-based predicted optical density (spectra + ML-model), and anticipated spectrum-based predicted optical density (spectra + anticipation + ML-model) for representative batches at multiple fermentation stages.

Across batches, consistent trends were observed, with predictions generated using a 10 h anticipation horizon showing a strong agreement with experimental measurements. As the prediction horizon increased, prediction accuracy progressively decreased. In particular, predictions generated 30 h in advance exhibited larger discrepancies between estimated and measured spectra at later fermentation stages. Nevertheless, the corresponding predicted optical density values remained in reasonable agreement with experimental measurements, indicating that longer-horizon predictions still provide a meaningful approximation of the system behavior. Similar results have been obtained by applying the same methodology to another platform based on CHO cells.

Regarding the choice of linear fitting for the extrapolation steps, the selection was driven by predictive performance rather than the visual quality of the fit alone. Although some accumulated trajectories may appear slightly non-linear over short intervals, the linear model consistently provided the most robust extrapolation behavior. In contrast, quadratic or cubic models could better adapt to local fluctuations in the observed spectra, but this also increased the risk of overfitting and reduced stability in the future domain. The objective of the method is therefore not to maximize the fit on the accumulated spectra themselves, but to ensure reliable anticipation of unseen spectra. For this reason, the linear configuration was retained because it provided the most reliable anticipation performance across batches, even if it did not always appear optimal from a visual standpoint.

To provide further perspective on these results, we also report as a comparison in Appendix A the performances (in RMSE) of the baseline naive approach and the proposed approach (Table A4 and Table A5). The reported RMSE values are calculated relative to the OD values predicted by the chemometrics model. As the anticipation horizon increases, both models exhibit increasing RMSE values.

Based on these results, subject matter experts for this particular application state that a 20 h prediction remains useful. For a fermentation process that typically runs for approximately 90 h, a 20 h prediction horizon offers a full day’s foresight into the culture’s trajectory. This timeframe is particularly valuable for two main reasons:

- *Early Assessment of Experimental Runs:* In a development setting, many runs are experimental, testing new process conditions. A 20 h prediction allows scientists to establish early assessments of whether a run is performing as expected. If the anticipated trajectory for key metrics (such as biomass) is highly unfavorable, it indicates that the experimental condition is not viable.
- *Resource Optimization:* Based on this predictive insight, a data-driven “go/no-go” decision can be made. By identifying and terminating unpromising fermentation runs approximately 24 h after their start, one can save significant resources—valuable operator time, media, and, most importantly, bioreactor capacity. This allows us to reallocate these resources to a new, more promising experimental run much sooner than if we had waited for the full 90 h process to complete. This holds for both development and commercial manufacturing contexts.

Therefore, even with a slight increase in prediction error (as indicated by $\Delta t_{min}$), the ability to make a well-informed decision to stop a failing batch a day in advance provides a substantial benefit, significantly accelerating the overall process development timeline and optimizing resources at several process lifecycle stages.

Another attractive element is that the method is designed to be highly responsive. A new spectrum is acquired every 5 min, feeding the model with near-real-time information. The prediction is generated based on a moving window of the 60 most recent spectra (5 h). In the event of a sudden process deviation or equipment failure, the model’s reaction would be as follows:

1. Initial Detection: The first spectrum acquired after the event (within 5 min) will be different from the previous ones. When this new spectrum enters the moving window, the prediction will immediately start to diverge from its previous trajectory, reflecting the change.
2. Full Adaptation Time: The prediction will become fully representative of the new process state once the moving window is completely refreshed with data post-event. Given that a spectrum is taken every 5 min and the window requires 60 spectra, the model will have fully adapted to the new conditions after 5 h (60 spectra * 5 min/spectrum).

Therefore, while there is a lag, the prediction is not static. It begins to adjust within minutes of a deviation. The “refresh rate” is inherently tied to the data acquisition frequency and the window size, resulting in a full system adaptation within approximately hours of a major, sustained event. This timeframe is considered acceptable for common monitoring purposes, as it still allows for timely intervention.

It is worth emphasizing that the limitation related to non-monotonous behaviors mainly becomes critical when considering long anticipation horizons. In practice, for short-term predictions (e.g., on the order of one hour), the method was observed to behave robustly, including for metabolites exhibiting non-monotonous dynamics. Such short-term anticipation already provides valuable operational insight, as it enables early detection of upcoming trends and supports timely decision-making during the fermentation process.

By combining runs with different process parameters ($pO_{2}$, feeding), we ensured that the final dataset was representative of a wide range of potential real-world conditions. The strong performance of our method across this entire, varied collection of lots confirms its robustness and its ability to generalize beyond a single, idealized process.

These results demonstrate the interest of the method in practice when combined with chemometrics models. Another practical benefit of the method is that it does not require any training or reference dataset to put the spectral anticipation methodology into practice. Each application just requires the first few hours of spectra to be produced for a given cell culture batch. This also offers a modular approach, where spectra anticipation is independent of the specificities of models, mechanistic or not, that could be used: any valid model that takes spectra as input may benefit from this methodology, in a “plug-and-play” spirit. Another appealing aspect related to the simplicity of the method is that, contrary to deep learning methods, it does not require high-performance computational hardware (no large CPU, and no GPU at all), making it fit for application in practice in regular industrial contexts.

Yet we are able to outline some limitations to the method and outline future work to overcome them. Although it is shown in this work that, in most cases, the first two components of the PCA are sufficient to capture around 99% of explained variance, one could adapt the method, if presented with more challenging datasets, to include a third component. In such a case, a similar investigation should occur to study the nature of the relation between PC1 + PC2 on one hand, and PC3 on the other hand. As was the case for the first component (with respect to time) and second component (with respect to the first) in a cross-validated framework, several degrees of complexity could be studied.

One limitation is that, by nature, the method copes better with nearly monotonous behaviors, as it relies on extrapolation. This nearly monotonous behavior is the one shown by biomass, for example. It only roughly increases over the course of a cell culture run. Biomass is a central metric to monitor, but some metabolites, such as ethanol, may present an increase, up to a peak, then a decrease. The current spectral anticipation method will be limited in managing the switch from increase to decrease, while it will perform well overall on the rest of the curve (respectively before and after this peak). This kind of non-monotonous behavior could be anticipated based on other methods which, contrary to the one introduced in this paper, have referenced historical datasets containing spectra corresponding to such courses, and could anticipate the occurrence of this switch.

## 5. Conclusions

This paper introduces and evaluates a method for Raman spectra anticipation in the context of bioproducts cell culture. The method relies on the extrapolation of future spectra from observed spectra in a lower-dimensionality space (two dimensions), obtained by a PCA. The extrapolated data is then back-projected to the original space of the spectra.

The method thus positions itself for use in contexts where chemometric models are available to perform monitoring based on concomitant spectra [10], as an extension providing them with the capability of predicting the future course of a cell culture. Those models may or may not, in turn, be used in conjunction with mechanistic models [20].

The method is evaluated qualitatively and quantitatively. It is also put in a concrete biotech context. These evaluations support the attractiveness of the method, which seems to be suitable for applications around 20 h of anticipation in a real industrial context. Another very appealing feature of the method is that it does not require a historical dataset to start implementing this method: there is no model “learning”/fitting required.

Spectra anticipation implements predictive monitoring and even paves the way for prescriptive monitoring. Systems implementing spectra anticipation, chemometrics monitoring models, and an extra regulation feedback loop (on feeding, typically), could achieve self-regulation, course correction and even yield optimization.

## Figures and Tables

**Table 1:** Mean and standard deviation of $\Delta t_{min}\left(i\right)$, in hours, for varying amounts of anticipation (*i*) with the spectra anticipation method.

*i* (h)	$\bar{\Delta t_{\min}\left(i\right)}$ ± sd 1 h	$\bar{\Delta t_{\min}\left(i\right)}$ ± sd 30 min
1	−0.19 ± 0.51	−0.19 ± 0.79
5	−0.25 ± 1.58	−0.42 ± 2.39
10	0.42 ± 5.04	0.17 ± 5.07
13	1.31 ± 7.50	1.44 ± 7.72
15	2.68 ± 10.03	2.15 ± 8.81
20	6.83 ± 14.55	5.72 ± 13.00
25	9.76 ± 16.74	8.62 ± 15.09
30	11.35 ± 16.62	7.72 ± 14.73

**Table 2:** Mean and standard deviation of $\Delta t_{min}\left(i\right)$, in hours, for varying amounts of anticipation (*i*), obtained with the naive baseline model.

*i* (h)	$\bar{\Delta t_{\min}\left(i\right)}$ ± sd 1 h	$\bar{\Delta t_{\min}\left(i\right)}$ ± sd 30 min
1	−1.00 ± 0.02	−1.00 ± 0.01
5	−5.02 ± 0.06	−5.00 ± 0.01
10	−10.04 ± 0.08	−9.99 ± 0.02
13	−13.05 ± 0.08	−12.99 ± 0.02
15	−15.05 ± 0.09	−14.99 ± 0.02
20	−20.05 ± 0.09	−19.98 ± 0.02
25	−25.06 ± 0.09	−24.98 ± 0.02
30	−30.07 ± 0.10	−29.97 ± 0.02

**Table 3:** Mean and standard deviation of $\Delta t_{min}\left(i\right)$, in hours, for varying amounts of anticipation (*i*), with prior d2 detrending of the spectra for the spectra anticipation method.

*i* (h)	$\bar{\Delta t_{\min}\left(i\right)}$ ± sd 1 h	$\bar{\Delta t_{\min}\left(i\right)}$ ± sd 30 min
1	−1.04 ± 0.31	−0.96 ± 0.86
5	−2.40 ± 2.92	−1.33 ± 3.89
10	−0.50 ± 6.51	−0.90 ± 6.71
13	2.02 ± 10.27	−0.08 ± 8.81
15	3.90 ± 12.60	0.71 ± 10.63
20	6.64 ± 15.74	2.81 ± 13.66
25	8.85 ± 16.84	3.91 ± 13.98
30	9.94 ± 16.49	3.38 ± 14.33

**Figure A1:**
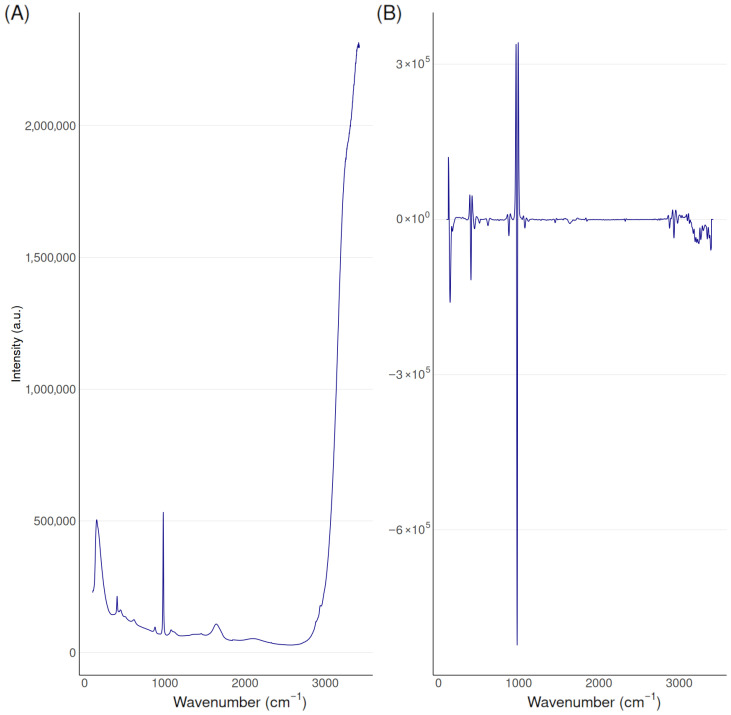
Comparison of different spectral representation. (**A**) Raw spectra. (**B**) Spectra processed using a second-derivative detrending method.

**Figure A2:**
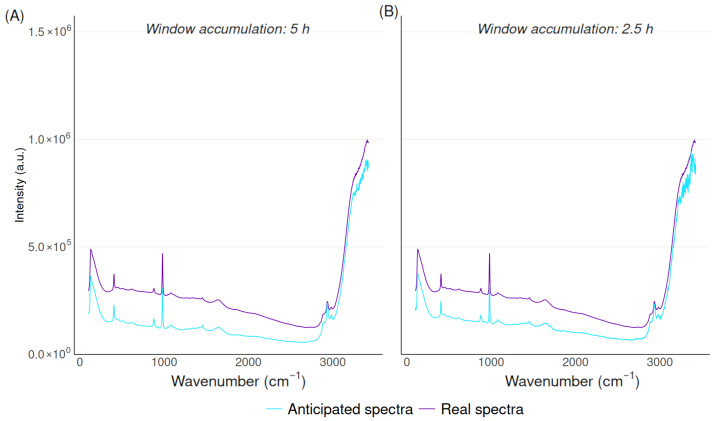
Comparison of anticipated spectra obtained with (**A**) 5 h and (**B**) 2.5 h accumulation windows.

**Figure A3:**
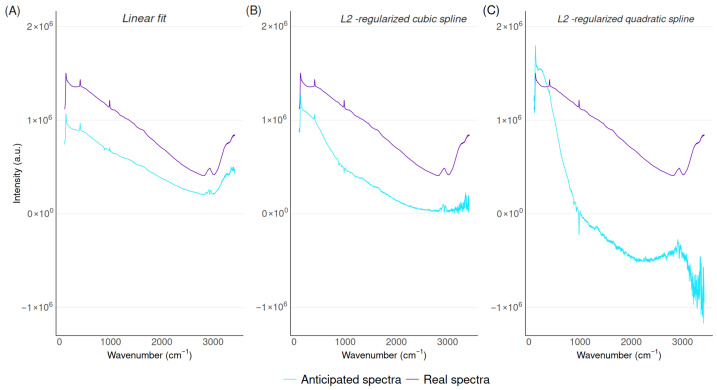
Comparison of spectra anticipation using (**A**) linear fit, (**B**) $L_{2}$-regularized cubic spline, and (**C**) $L_{2}$-regularized quadratic spline for PC2 vs. PC1.

**Figure A4:**
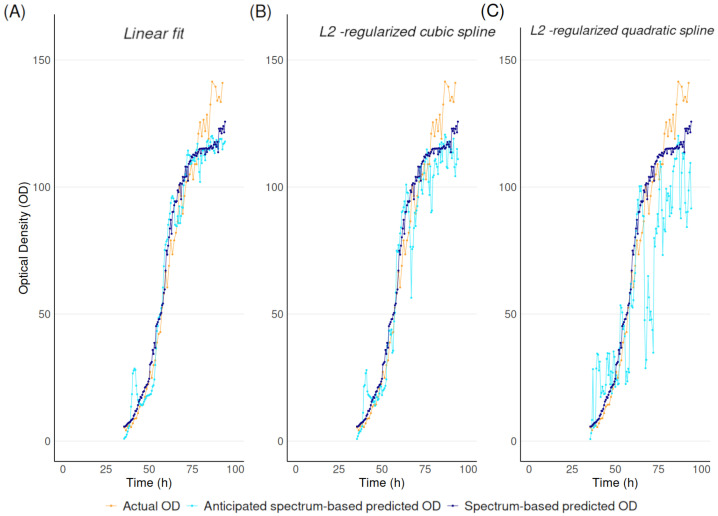
Comparison of OD predictions using (**A**) linear fit, (**B**) $L_{2}$-regularized cubic spline, and (**C**) $L_{2}$-regularized quadratic spline for PC2 vs. PC1.

**Figure A5:**
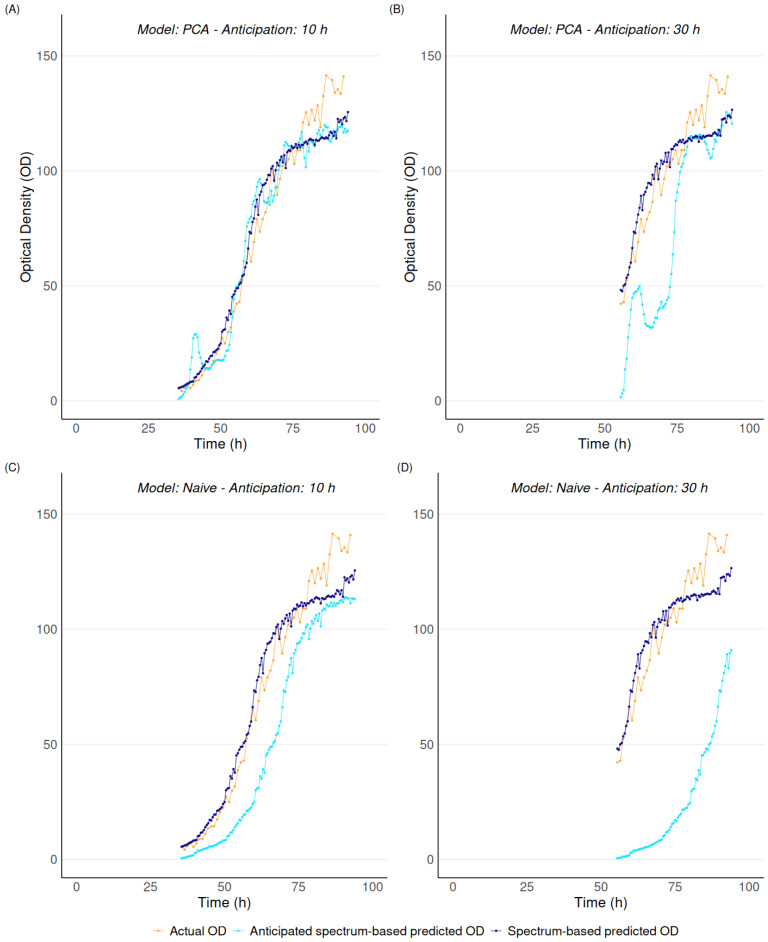
Comparison of actual OD, spectrum-based predicted OD, and anticipated spectrum-based predicted OD for two models (PCA and naive) and two anticipation horizons (10 h and 30 h). (**A**) PCA model, 10 h anticipation. (**B**) PCA model, 30 h anticipation. (**C**) Naive model, 10 h anticipation. (**D**) Naive model, 30 h anticipation.

## Data Availability

The raw data supporting the conclusions of this article will be made available by the authors on request.

## Abbreviations

The following abbreviations are used in this manuscript:

DOAJ	Directory of open access journals.
MPC	Model Predictive Control.
MDPI	Multidisciplinary Digital Publishing Institute.
PAT	Process Analytical Technology.
PCA	Principal Component Analysis.
RMSE	Root Mean Squared Error.

## Appendix A

### Appendix A.1. Comparison of Alternative Methodological Choices

Before converging to the methodology reported in Section 2.2, several alternative configurations of the spectra anticipation pipeline were evaluated. In particular, three primary configuration parameters were combined: start time, wavelength range, and spectrum preprocessing. Their combinations resulted in 2×2×3=12 distinct strategies. All these strategies performed less well than or equivalently to the one presented in Section 2.2, which led to the results reported in Section 3.

#### Appendix A.1.1. Primary Configuration Parameters

Three parameters define the general configuration of the anticipation method:

- *Start time.* The initial period of spectra accumulation before starting to anticipate new spectra was investigated. Two alternatives were compared: starting accumulation immediately at fermentation initiation (0 h) or delaying the start to 20 h. This question arose from the fact that, in general, the first few hours of cell culture lead to very low measurements, sometimes below the limit of detection, for various quantities, and can thus be assimilated to noise (we refer here to noise in the offline measurements, below the limit of detection, not noise in the corresponding Raman spectra).
- *Wavelength range.* We compared three alternatives, including one where the complete spectra are used on all available wavelengths to produce estimations of future spectra. The second method is similar, but first truncates the wavelengths smaller than 100 cm^−1^ and larger than 3000 cm^−1^ before applying the same anticipation method. The third focuses on specific, much-reduced bands of wavelength specifically used by given chemometrics models, and thus truncates all wavelengths that are not in these bands before applying the anticipation method.
- *Spectrum preprocessing.* The anticipation method has been evaluated on both raw spectra and spectra processed with a second-derivative detrending method Figure A1. This consideration arises from the fact that such detrending is a common practice for chemometrics models training, but its impact on our current approach was not known in advance. Figure A1.

#### Appendix A.1.2. Refinement Parameters

In addition to these twelve strategies defined by the primary configuration parameters, three refinement parameters were adjusted to improve prediction stability and practical performance:

- *Spectra accumulation window.* Two accumulation window sizes were tested: 5 h (selected) and 2.5 h. When the 2.5 h window was used, predicted spectra became noticeably noisier compared to the 5 h window, as shown in the Figure A2. Predictions of optical density (OD) using the 2.5 h window showed increased variability around the actual trajectory. The 5 h window was retained as the optimal balance between data accumulation and prediction stability.
- *PC1 versus time fitting.* The relationship between PC1 and time was found to be highly linear across batches’ anticipation horizons, with coefficients of determination consistently close to 1, ranging from 0.96 to 0.98. As a consequence, only a linear fit of PC1 versus time was implemented and retained in the final configuration.
- *PC2 versus PC1 fitting.* Three fitting approaches were compared for the PC2 versus PC1 relationship: linear fit, $L_{2}$-regularized cubic spline, and $L_{2}$-regularized quadratic spline. As shown in Figure A3, spline-based and quadratic fittings yielded anticipated spectra that were noticeably noisier than those obtained with the linear model. This increased spectral noise propagated to the downstream OD predictions, resulting in less stable and more irregular OD curves. In contrast, linear fitting of PC2 versus PC1 produced smoother anticipated spectra and consequently smoother predicted OD curves, with reduced noise (Figure A4). For these reasons, the linear model was retained in the optimal configuration.

#### Appendix A.1.3. Extra Comparisons and Performance Evaluations

To evaluate the quality of spectral reconstruction, we compared the predicted spectra obtained from two approaches: PCA-based reconstruction and a naive baseline against the corresponding reference spectra, for both 1 h and 30 min acquisition frequencies. Performance was assessed using two complementary metrics: the Pearson correlation, which quantifies spectral shape similarity, and the root mean square error (RMSE), which measures absolute reconstruction error.

Across both acquisition settings, the PCA-based approach consistently outperformed the naive baseline for all anticipation times and for both evaluation metrics. The PCA model systematically achieved higher Pearson correlation values, indicating better preservation of spectral structure, while also producing lower RMSE values, reflecting improved quantitative reconstruction accuracy.

In contrast, the naive approach exhibited a clear degradation in performance as the anticipation horizon increased, with progressively lower correlation coefficients and substantially higher reconstruction errors. This trend was observed for both sampling frequencies, although the degradation was more pronounced at longer anticipation times.

Turning to the application of the method in conjunction with the chemometrics model, we compared the performances of the PCA-based proposed anticipation method and the naive baseline approach in predicting the OD of cultures. As can be seen from Table A4 and Table A5, the PCA-based anticipation model, which is more sophisticated, provides more accurate predictions than the naive model, showing a lower RMSE. These findings emphasize the practical limitations of the naive model, particularly for more complex tasks requiring future predictions based on evolving spectra.

To further assess the practical relevance of the anticipation method, we compare optical density (OD) predictions obtained from anticipated spectra using both the PCA-based model and a naive baseline for two anticipation horizons (10 h and 30 h). The results for a representative batch are shown in Figure A5.

**Table A1:** Comparison of predicted spectra against reference spectra using PCA-based reconstruction and a naive baseline for a 30 min acquisition frequency. Performance is evaluated using Pearson correlation and RMSE. Values are reported as mean and standard deviation.

Anticipation Time (h)	Pearson (PCA)	Pearson (Naive)	RMSE (PCA)	RMSE (Naive)
1	1 ± 0	1 ± 0	1.90 × $10^{4}$ ± 1.14 × $10^{4}$	2.23 × $10^{4}$ ± 6.28 × $10^{3}$
5	0.99 ± 0.01	0.98 ± 0.01	6.98 × $10^{4}$ ± 4.64 × $10^{4}$	1.04 × $10^{5}$ ± 2.68 × $10^{4}$
10	0.97 ± 0.02	0.92 ± 0.02	1.36 × $10^{5}$ ± 8.41 × $10^{4}$	1.96 × $10^{5}$ ± 4.39 × $10^{4}$
13	0.94 ± 0.04	0.88 ± 0.02	1.75 × $10^{5}$ ± 9.11 × $10^{4}$	2.54 × $10^{5}$ ± 5.50 × $10^{4}$
15	0.92 ± 0.04	0.84 ± 0.03	2.04 × $10^{5}$ ± 9.39 × $10^{4}$	2.93 × $10^{5}$ ± 6.34 × $10^{4}$
20	0.81 ± 0.06	0.72 ± 0.05	2.88 × $10^{5}$ ± 8.22 × $10^{4}$	3.93 × $10^{5}$ ± 8.89 × $10^{4}$
25	0.67 ± 0.07	0.58 ± 0.07	3.86 × $10^{5}$ ± 9.81 × $10^{4}$	4.90 × $10^{5}$ ± 1.12 × $10^{5}$
30	0.57 ± 0.08	0.42 ± 0.09	4.69 × $10^{5}$ ± 9.47 × $10^{4}$	5.87 × $10^{5}$ ± 1.36 × $10^{5}$

**Table A2:** Comparison of predicted spectra against reference spectra using PCA-based reconstruction and a naive baseline for a 1 h acquisition frequency. Performance is evaluated using Pearson correlation and RMSE. Values are reported as mean and standard deviation.

Anticipation Time (h)	Pearson (PCA)	Pearson (Naive)	RMSE (PCA)	RMSE (Naive)
1	1 ± 0.01	0.99 ± 0.01	1.27 × $10^{4}$ ± 8.13 × $10^{3}$	2.65 × $10^{4}$ ± 7.56 × $10^{3}$
5	0.99 ± 0.01	0.97 ± 0.01	4.21 × $10^{4}$ ± 1.07 × $10^{4}$	1.13 × $10^{5}$ ± 1.41 × $10^{4}$
10	0.98 ± 0.01	0.91 ± 0.02	1.03 × $10^{5}$ ± 1.64 × $10^{4}$	2.27 × $10^{5}$ ± 2.63 × $10^{4}$
13	0.95 ± 0.02	0.86 ± 0.03	1.50 × $10^{5}$ ± 2.10 × $10^{4}$	2.97 × $10^{5}$ ± 3.33 × $10^{4}$
15	0.92 ± 0.03	0.82 ± 0.04	1.87 × $10^{5}$ ± 2.53 × $10^{4}$	3.44 × $10^{5}$ ± 3.78 × $10^{4}$
20	0.81 ± 0.04	0.71 ± 0.06	2.96 × $10^{5}$ ± 4.01 × $10^{4}$	4.64 × $10^{5}$ ± 4.83 × $10^{4}$
25	0.7 ± 0.03	0.58 ± 0.07	4.18 × $10^{5}$ ± 5.25 × $10^{4}$	5.86 × $10^{5}$ ± 5.85 × $10^{4}$
30	0.61 ± 0.05	0.45 ± 0.08	5.48 × $10^{5}$ ± 6.37 × $10^{4}$	7.07 × $10^{5}$ ± 6.93 × $10^{4}$

**Table A3:** Mean and standard deviation of $\Delta t_{min}\left(i\right)$, in hours, for varying amounts of anticipation (*i*), obtained with the naive baseline model after d2 detrending of the spectra.

*i* (h)	$\bar{\Delta t_{\min}\left(i\right)}$ ± sd 1 h	$\bar{\Delta t_{\min}\left(i\right)}$ ± sd 30 min
1	−1.00 ± 0.02	−1.00 ± 0.01
5	−5.02 ± 0.06	−5.00 ± 0.01
10	−10.04 ± 0.08	−9.99 ± 0.02
13	−13.05 ± 0.08	−12.99 ± 0.02
15	−15.05 ± 0.09	−14.99 ± 0.02
20	−20.05 ± 0.09	−19.98 ± 0.02
25	−25.06 ± 0.09	−24.98 ± 0.02
30	−30.07 ± 0.10	−29.97 ± 0.02

**Table A4:** RMSE for optical density (OD) prediction using chemometrics model, PCA-based anticipation, and naive baseline, for 1 h acquisition frequency. Values are reported as mean (5th percentile–median–95th percentile).

Anticipation Time (h)	Chemometrics	PCA	Naive
1	10.4 (6.79–9.85–14.21)	10.77 (6.86–10.09–15.19)	10.96 (6.02–10.23–16)
5	10.61 (6.8–10.66–13.77)	12.89 (9.01–13.05–16.18)	14.64 (5.79–15.12–22.68)
10	11.05 (7.48–10.92–14.26)	15.01 (8.21–14.78–24)	23.52 (9.64–23.9–32.62)
13	11.15 (7.01–11.17–14.3)	15.87 (9.61–16.31–23.5)	29.91 (14.73–31.39–39.29)
15	11.65 (7.64–11.43–14.79)	15.19 (8.71–16.22–22.39)	34.18 (16.94–36.22–44.07)
20	12.08 (8.18–11.9–15.05)	16.93 (10.42–16.94–24.07)	45.46 (24.93–49.69–57.8)
25	12.56 (8.05–12.51–15.74)	21.2 (12.74–21.16–31.08)	57.33 (33.42–62.56–72.24)
30	12.92 (8.31–13.36–16.44)	26.66 (15.98–27.23–36.79)	68.35 (39.05–73.48–86.55)

**Table A5:** RMSE for optical density (OD) prediction using chemometrics model, PCA-based anticipation, and naive baseline, for 30 min acquisition frequency. Values are reported as mean (5th percentile–median–95th percentile).

Anticipation Time (h)	Chemometrics	PCA	Naive
1	8.61 (5.66–7.46–13.09)	8.85 (6.33–7.81–12.53)	9.33 (6.87–7.42–13.76)
5	8.89 (5.84–8.18–13.07)	9.9 (6.91–10.64–12.32)	16.01 (11.22–16.29–20.93)
10	9.26 (6.15–8.05–13.43)	13.25 (9.82–14.22–15.32)	28.1 (22.57–29.68–32.01)
13	9.35 (6.14–8.44–13.52)	14.85 (11.87–15.53–16.87)	35.3 (29.75–36.97–39)
15	9.29 (5.81–8.62–13.93)	14.93 (12.47–15.77–17.06)	40.15 (34.75–41.54–44.27)
20	9.64 (6.23–8.66–14.3)	17.87 (14.28–19.26–21.63)	53.9 (48.51–55.54–58)
25	10.08 (6.43–9.35–14.78)	23.92 (14.97–24.86–32.08)	67.23 (62.35–67.68–70.68)
30	10.01 (6.62–9.4–13.85)	30.79 (17.24–31.79–44.62)	80.83 (76.13–81.98–83.23)

For a 10 h anticipation horizon, both models are able to capture the overall growth dynamics of the culture. However, the PCA-based approach (A) provides predictions that remain closely aligned with the spectrum-based OD estimates and the actual measurements throughout the growth phase. In contrast, the naive model (C) exhibits a noticeable temporal lag, particularly during the exponential growth phase, leading to a systematic underestimation of OD.

When increasing the anticipation horizon to 30 h, the differences between the two approaches become more pronounced. The PCA-based model (B) still captures the general trend of the growth curve, although with some degradation in accuracy, especially during rapid transitions. On the other hand, the naive model (D) fails to reproduce the correct dynamics, with strong delays and significant deviations from both the actual and spectrum-based predictions.

Overall, these results demonstrate that the PCA-based anticipation method provides more robust and temporally consistent predictions, particularly as the anticipation horizon increases. This highlights its ability to better capture the underlying dynamics of spectral evolution compared to the naive approach, which remains limited in its capacity to extrapolate complex temporal patterns.
